# Assessment of Sclerostin and Interleukin 6 Levels and Selected Anthropometric Parameters in Patients Receiving Hemodialysis Replacement Therapy—Pilot Study

**DOI:** 10.3390/medicina55120784

**Published:** 2019-12-15

**Authors:** Agnieszka Turon-Skrzypinska, Grazyna Dutkiewicz, Malgorzata Marchelek-Mysliwiec, Violetta Dziedziejko, Kazimierz Ciechanowski, Aleksandra Ryl, Iwona Rotter

**Affiliations:** 1 Department of Medical Rehabilitation and Clinical Rehabilitation, Pomeranian Medical University, 71-210 Szczecin, Poland; ryl.ola@gmail.com (A.R.); iwrot@wp.pl (I.R.); 2 Department of Nephrology, Transplantology and Internal Medicine, Pomeranian Medical University, 70-111Szczecin, Poland; grazyna_dutkiewicz@o2.pl (G.D.); malgorzata.marchelek@gmail.com (M.M.-M.); kazcie@pum.edu.pl (K.C.); 3 Department of Biochemistry and Medical Chemistry, Pomeranian Medical University, 70-111 Szczecin, Poland; viola@pum.edu.pl

**Keywords:** chronic kidney disease, sclerostin level in hemodialysis patients, interleukin 6 level in hemodialysis patients, anthropometric parameters

## Abstract

*Background and Objectives*: Chronic kidney disease (CKD) is an important public health problem associated with, e.g., progressive renal insufficiency, bone mineral disorders, and increased inflammatory marker levels. The objective of this study was to compare selected biochemical parameters and to evaluate potential correlations between selected anthropometric parameters and levels of sclerostin and interleukin 6 (IL-6) in blood plasma. *Materials and Methods*: The study group consisted of 34 patients aged 59.8 ± 9.8 years, receiving hemodialysis therapy. The control group consisted of 31 individuals aged 55.4 ± 9.37 years, presenting with GFR (glomerular filtration rate) of more than 60 mL/min/1.73 m^2^. Selected anthropometric and biochemical parameters were assessed at baseline, as well as 3 and 6 months into the study. Statistical analyses were performed using the Statistica 2014 software package (StatSoft, Inc.Tulsa, OK, USA). Analyses included descriptive statistics, intergroup comparisons using the Mann-Whitney U-test or the Kruskal-Wallis test, and Spearman’s correlation analysis. The significance level was set at *p* ≤ 0.005. *Results*: At all measurement time points, i.e., at baseline, at month 3, and at month 6, the IL-6 levels in the study group were significantly higher than those in the control group. No correlations were observed in the study group between SCL or IL-6 levels and anthropometric parameters such as body weight, body mass index (BMI), or waist circumference. *Conclusions*: Patients receiving hemodialysis replacement therapy present with significantly higher levels of IL-6 in their blood. Anthropometric parameters (body weight, BMI, and waist circumference) have no impact on sclerostin and IL-6 levels in patients undergoing hemodialysis therapy. The results obtained are satisfactory, and the research will be continued.

## 1. Introduction

Chronic kidney disease (CKD) is classified, along with arterial hypertension, cardiovascular disorders, diabetes, and obesity, as one of the lifestyle diseases, which pose significant problems to public health [1]. It affects about 600 million individuals worldwide, including about 4 million individuals in Poland. As of today, hemodialysis is the most common modality of renal replacement therapy (RRT) in patients with end-stage renal disease [2,3]. Patients undergoing hemodialysis therapy frequently experience chronic kidney disease-mineral bone disorders (CKD-MBD) and present with increased levels of inflammatory markers in blood [3].

Sclerostin, a 28 kDa glycoprotein encoded within chromosome 17 (region 17q12-q21) in the vicinity of the SOST gene, plays an important role in bone mass regulation and the inhibition of anabolic bone formation via attenuation of osteoblast differentiation [4,5,6]. Increased sclerostin levels (SCL) are first observed at stage III CKD. In patients with end-stage renal disease, sclerostin concentrations are several-fold higher than those in subjects with normal renal function [7,8,9,10,11,12]. Bone metabolism disorders may result in blood vessel calcifications, atherosclerosis, or myocardial ischemia [13]. Therefore, periodic monitoring of SCL in CKD patients may be of significant diagnostic importance with regard to bone turnover. 

CKD is also associated with increased concentrations of proinflammatory cytokines, including interleukin 6 (IL-6, a 26 kDa protein encoded by a gene located within the 7p15-p21 region of the short arm of chromosome 7) [14,15,16]. Upon inflammation, IL-6 levels may be increased by a factor of up to 100. IL-6 is mostly subject to renal and hepatic elimination [17]. In patients with end-stage renal disease, the increase in proinflammatory cytokine levels is also due to protein insufficiencies. Moreover, reduced muscle clearance leads to the systemic accumulation of these cytokines [18]. CKD patients are at risk of numerous infections due to exposure to bacteria or viruses. Inflammation is also promoted by the presence of foreign bodies, hemodialysis catheters, arteriovenous fistulas, or the hemodialysis procedure itself [18]. Elevated cytokine levels are also due to chronic stress and negative emotions [17,19,20].

To our knowledge, single comparative studies on the association of serum SCL with selected biochemical and anthropometric characteristics in patients undergoing maintenance hemodialysis have been conducted. Further studies are needed to determine these associations.

The objective of this study was to assess selected anthropometric and biochemical parameters, including SCL and IL-6 levels, in the course of hemodialysis replacement therapy and to evaluate potential correlations between selected anthropometric parameters and plasma levels of sclerostin and IL-6 in patients receiving hemodialysis replacement therapy.

## 2. Materials and Methods

The study included 34 end-stage renal disease patients undergoing hemodialysis renal replacement treatment due to the complete absence of diuresis. Patients in the study group received three hemodialysis sessions per week, with the average duration of one session amounting to 242.6 ± 25.2 min (X ± SD). The mean duration of hemodialysis therapy of the patients in the study group was 56 months. 

The control group (31 subjects) consisted of subjects presenting at follow-up visits at a family physician office. These outpatients were attending an appointment and were screened for renal function. Blood samples were collected for blood count from all participants, and analyses to determine serum levels of creatinine, urea, total proteins level, and urine were performed. Patients with eGFR of above 60 mL/min/1.73 m^2^ were included in the study control group. A total of 31 subjects were randomly selected for the control group.

The study included patients on the basis of inclusion and exclusion criteria. Subjects with CKD aged over 18 years with anuria, undergoing maintenance hemodialysis from at least 3 months, three times per week were enrolled in the study group. Subjects with visual impairment not subjected to treatment, and those with senile dementia or depression, as well as poorly-controlled diabetes with HbA_1_C levels of over 8%, were not enrolled in the study. Patients with severe cardiovascular diseases, malignant tumors, and musculoskeletal disease were not recruited. Similar excluding criteria were used in the control group. The study protocol was described to all patients, and each participant provided informed consent.

Overall, the study population consisted of 65 subjects. The study group consisted of 13 (38.2%) females and 21 (61.8%) males, whereas the control group consisted of 15 (48.4%) females and 16 (51.6%) males. No statistically significant differences were observed between the groups in terms of patient gender, age, body weight, body mass index (BMI), and waist circumference (Table 1). All participants declared no other serious chronic disease. The number of hospitalizations was significantly higher in the study group as compared to the control group.

### 2.1. The Course of the Study

The study was approved by the Bioethics Committee decision no. KB-0012/40/13(2013-03-04). The study was designed as a prospective cohort trial. Data were collected at three time points: upon study inclusion (E0), after 3 months of study (E3), and after 6 months of study (E6).

The patients’ task consisted of filling out a proprietary survey questionnaire containing demographic questions designed to collect information on the health status and lifestyle of patients.

Selected anthropometric measurements of patients were taken at three time points, i.e., at E0 (baseline measurement at the start of the study), E3 (3 months into the study), and E6 (6 months into the study) with the exception of height, which was measured at E0 only. In the study group, measurements were taken 20–30 min after completion of hemodialysis in an appropriately fitted examination room. Patient height was measured vertically from the floor to the head vertex using a height meter integrated with an electronic balance. Body weight measurements were taken using the same balance with an accuracy of ±10 g. Waist circumferences were measured using a centimeter-scale body measuring tape. Weight/stature proportions were assessed using the BMI [21,22].

A total of 2 mL of blood was collected in EDTA(ethylenediaminetetraacetic acid) tubes (patients with end-stage renal disease had blood taken from the venous line of the dialysis shunt before the HD procedure) from the study group and the control group during periodic follow-up examination visits at E0, E3, and E6 at the dialysis unit of the Department of Nephrology, Transplantology, and Internal Medicine, Pomeranian Medical University. Samples were centrifuged (4000 rpm for 10 min) at 4°C using an MPW-350R centrifuge. Plasma samples were divided into 1.0-mL aliquots placed in two separate 1.0-mL Eppendorf Quality™ Safe-Lock tubes (colorless) (Eppendorf, Warsaw Poland) and immediately frozen. Samples were stored at −170°C until the time of analysis. Laboratory analyses were performed on freshly thawed portions. Plasma levels of IL-6 and sclerostin were determined using a commercially available Quantikine High Sensitivity ELISA kit (R&D Systems Europe, Abingdon, UK). SCL and IL-6 level were measured in picograms per milliliter [pg/mL]. 

Dry body weight was estimated empirically on the basis of a clinical examination (presence of clinical features of overhydration) and on the basis of ultrasound assessment of the diameter of the inferior vena cava (IVC) and its changes in various respiratory phases [22].

### 2.2. Statistical Analysis

All statistical computations were performed using a StatSoft (Inc. Tulsa, OK, USA) STATISTICA data analysis software system version 12.0 (2014, www.statsoft.com) and MS Excel spreadsheets. 

Quantitative variables were characterized by arithmetic means, standard deviations, as well as median, minimum, and maximum values. Qualitative variables were characterized by absolute counts and percentages. Intergroup differences were assessed using the Mann-Whitney U-test, whereas correlations between parameters were assessed using Spearman’s rank correlation coefficient (Rho). Correlation coefficients or Spearman’s rank coefficients were used to determine the strengths and directions of correlations between variables. For all computations, the significance level was set at *p* ≤ 0.05.

## 3. Results

The study group and the control group were compared in terms of SCL and IL-6 level in plasma (Table 2). No significant differences were detected between SCL levels in the patients in the study group and the control group at individual time points. In contrast, the levels of IL-6 were significantly higher in the patients in the study group than those in the subjects in the control group at all stages of the study.

Correlations were also assessed between selected anthropometric parameters and the SCL and IL-6 levels in the study group and control group at baseline and at months 3 and 6 (Table 3). In the study group, no correlation could be found between SCL levels and the assessed parameters. In the control group, increasing SCL levels were associated with increasing waist circumference at baseline and at months 3 and 6, and a positive correlation was found between SCL at month 3 and the body weight and BMI values. In addition, we observed a relationship between sclerostin serum levels and WHR in each of the analyzed measurement. 

Increasing IL-6 levels in the control group were associated with increasing body weight, BMI, WHR, and weight circumference at months 3 and 6. No statistically significant correlations were identified for other relationships.

In the study group and the control group, the age of patients was correlated with increasing SCL levels at month 3 (Table 4). No statistically significant correlations were identified for other relationships.

Increasing IL-6 levels were observed with increasing age of the subjects in the control group. No statistically significant correlations were identified for other relationships.

The study also analyzed the relationship between the assessed parameters at individual measurement time points (E0, E3, and E6). In the control group, there was no relationship between individual measurements: BMI (*p* = 0.23588), WHR (*p* = 0.86688), waist circumference (*p* = 0.51342), hip circumference (*p* = 0.36788), SCL (*p* = 0.65297), and IL-6 (*p* = 0.50232). In contrast, in the study group, statistical relationships between the tested parameters were observed over time: BMI (*p* = 0.01414), waist circumference (*p* = 0.02001), hip circumference (*p* = 0.00219), and SCL (*p* = 0.01103). No relationship was observed in the analysis of the levels of IL-6 (*p* = 0.75872) and WHR (*p* = 0.62189).

## 4. Discussion

Anthropometric measurements are widely used in clinical studies. The most common measurements include body weight, height, and waist circumference [19]. In patients requiring hemodialysis, the analysis of BMI values is difficult due to overhydration. The use of the dry mass of hemodialyzed patients to calculate the index is more reliable, similar to the case in this study [21,23,24]. In this study, the analysis of selected anthropometric parameters revealed no statistically significant differences in body weight, waist circumference, and BMI values between the study and control groups. Burton et al. demonstrated a correlation between the risk of CKD and the BMI value or waist circumference [25]. In their study on CKD patients, Ishimura et al. found a gradual drop in dry body weight during the first year of hemodialysis therapy [26]. 

Numerous studies are available on the relationship between SCL and bone turnover parameters. Sclerostin deficiency is positively correlated with high bone mass. This means that disorders such as van Buchem disease or sclerosteosis are associated with a significant increase in bone mass density [27]. On the basis of the physiological role of SCL resulting in bone mass loss, SCL levels were shown to be correlated with body mass loss in HD patients [28]. Since SCL inhibits bone formation and promotes bone resorption, SCL levels should be positively correlated with bone resorption markers and negatively correlated with bone formation markers. However, numerous studies failed to confirm this mechanism because of the lack of appropriate correlations [29,30,31,32]. Observations regarding the role of SCL in the bone metabolism of both healthy subjects and CKD patients revealed that higher SCL levels were associated with higher bone mass. Our measurements taken at baseline, as well as after 3 and 6 months of study, revealed no differences between the study group and the control group in terms of SCL. Correlations of SCL levels in CKD patients as compared to subjects with no kidney disorders can be identified by a detailed analysis of the available literature. Kanbay et al. demonstrated that SCL levels in CKD patients were higher than those in subjects with normal kidney function [33]. In patients receiving RRT, blood SCL levels are significantly higher than those in individuals presenting with GFR of more than 60 mL/min [4,9,28,31,33,34]. As also shown by available studies, patients with advanced CKD present with increased blood SCL. Maximum values are observed in hemodialyzed patients [9,29,35]. These may be due to either kidney dysfunction or increased SCL production. However, no explanation of the mechanism behind increased SCL production has been provided yet. It has been suggested that it may result from hyperphosphatemia, osteocytic resistance to the effects of PTH, impaired binding of SCL by its specific receptors, or increased extraskeletal production [34,36,37,38,39,40,41,42]. Calcium and phosphorus metabolism disorders in the course of CKD are known to result in calcification of blood vessels [33]. However, vessel calcification may be influenced by different factors such as age, hypertension, diabetes, inflammation, or increased blood SCL [4,42]. Similar studies on the level of sclerostin in CKD patients were performed by Hamada-Ode et al., who indicated that the level of SCL is higher in CKD compared to healthy controls. They also showed that this concentration depends on the stage of kidney disease [43].

In this study, blood SCL levels were examined in terms of their correlations with selected anthropometric parameters (age, body weight, waist circumference, and BMI). In the study group, no significant correlation could be found between SCL levels and the assessed anthropometric parameters. In contrast, an increase in SCL was observed along with the increasing waist circumference at all measurement points. Also, a positive correlation was shown in the control group between the SCL levels at month 3 and the body weight and BMI values. The results of this research project may suggest that CKD and RRT disturb the assessed parameters. Sato et al. and Jean et al. observed a positive correlation between SCL levels and BMI values in hemodialyzed patients [11,38]. Pelletier et al. demonstrated a positive correlation between SCL levels and BMI values in a group of nonhemodialyzed CKD patients [38]. A positive correlation between SCL levels and BMI values was also observed in other disorders [39,40]. In addition, numerous publications examined correlations between SCL levels and age in various nosocomial entities [39,44,45,46]. There are also studies that show a reduction in the concentration of SCL levels during HD RRT [47].

Hemodialysis procedures may contribute to the development or exacerbation of inflammation due to impurities within the dialyzate, reaction with the dialysis membrane, or vascular access-related infections [48,49,50]. In our study, IL-6 was analyzed as the inflammation marker. IL-6 levels were shown to be significantly higher in HD (hemodialysis) patients as compared to the subjects in the control group with no CKD at all measurement points. Similar results were reported by other authors [51,52,53,54]. Most likely, these are due to continuous inflammation exacerbated by the hemodialysis procedure. Periodic hemodialysis escalates chronic inflammation. Pupim et al., who examined IL-6 levels in patients with end-stage renal disease, observed no changes in concentrations of this cytokine following initiation of RRT [55]. Published studies on the impact of single dialysis procedures on the levels of IL-6 in blood revealed that IL-6 concentration increases 2 h after the completion of hemodialysis, most probably as a response to RRT [48]. Likewise, Goldstein et al. suggested that high doses of hemodialysis significantly reduce baseline levels of IL-6 in children subjected to the procedure, with an increase in IL-6 levels being observed 2 h after the completion of the procedure [56]. Kato et al. and Wanic-Kossowska and Pawilczak demonstrated that IL-6 levels were positively correlated with the total duration of RRT [57,58]. Blood IL-6 levels are significantly higher in patients with depression; at the same time, CKD is frequently accompanied by depressed mood [59,60]. One may conclude that in the group of dialyzed patients, concentrations of IL-6 may be additionally increased by reduced mental health status. 

A study conducted to examine renal parameters revealed a positive correlation between IL-6 and BMI or body weight, while no significant correlation was observed between IL-6 levels and waist circumference [61]. This study revealed no correlations between body weight, waist circumference, or BMI and blood IL-6 levels in the study group. In contrast, baseline, month 3, and month 6 assessments in the control group revealed a positive correlation between the anthropometric parameters and IL-6 levels. As reported by other authors in their studies conducted on adult populations, significant correlations were observed between anthropometric parameters such as body weight or BMI and blood interleukin-6 levels [62]. In contrast, Park et al. confirmed a positive correlation between BMI or waist circumference and blood IL-6 levels in a group of patients without chronic disorders [63]. The available literature demonstrates correlations between the levels of IL-6 in peripheral blood and patient age [64,65]. In this group, a similar correlation was observed in the control group. The absence of correlation between IL-6 levels and patient age in the study group may be due to the overall health of CKD patients.

## 5. Conclusions

Patients receiving hemodialysis replacement therapy present with significantly higher levels of IL-6 in their blood. Anthropometric parameters (body weight, BMI, and waist circumference) have no impact on SCL and IL-6 level in patients undergoing hemodialysis therapy. 

### Limitations

This pilot study provides an excellent starting point for a broader prospective study to be conducted on a larger patient population. The study group was difficult to form due to the long study duration, kidney transplants, and death of patients with end-stage renal disease. Further study requires a larger study population for a more detailed analysis of correlations of interest. The inclusion of other inflammatory markers along with a more precise characterization of calcium and phosphate metabolism disorders, as well as other hormonal disorders accompanying chronic hemodialysis therapy and CKD, should also be considered. Another important aspect of future studies would be patient diet being controlled for the duration of the study. 

## Figures and Tables

**Table 1:** Basic characteristics of the study group and the control group.

Parameter	Study Group (*n* = 34)			Control Group (*n* = 31)			*p*-Value
	X (SD)	Range	Me	X (SD)	Range	Me	
**Age [years]**	59.8 (9.8)	29.0–78.0	60.5	55.4 (9.37)	35.0–73.0	59.0	0.1269
**Body weight [kg]**	74.6 (14.5)	64.5–81.5	75.4	78.0 (16.03)	65.8–91.3	79.0	0.45012
**BMI [kg/m^2^]**	25.8 (4.4)	22.1–28.8	25.3	26.7 (4.1)	24.1–29.8	25.9	0.33432
**Waist circumference [cm]**	96.8 (14.7)	88–105	96.5	91.4 (16.3)	83–100	89.0	0.06752
**Hip circumference [cm]**	101.7 (10.5)	73.0–123.0	102.0	102.5 (7.5)	91.0–120.2	103.0	0.8541
**WHR**	0.9 (0.1)	0.7–1.1	0.9	0.8 (0.1)	0.7–1.1	0.9	0.0108 *
**Hospitalizations**	2.9 (2.7)	0.0–15.0	2.5	0.2 (0.5)	0.0–3.0	0.0	0.00012 *

BMI—body mass index; WHR—waist hip ratio; X—arithmetic mean; SD—standard deviation; Me—median; range—(minimum–maximum); *p*-value—statistical significance; *— statistically significant parameter; *n*—number.

**Table 2:** Comparison of the study group and the control group in terms of sclerostin and IL-6 concentrations in plasma at three measurement points (baseline, month 3, month 6).

Variable	Observation Period	Study Group (*n* = 34)			Control Group (*n* = 31)			*p*-Value
		X (SD)	Range	Me	X (SD)	Range	Me	
**Sclerostin [pg/mL]**	E0	548.1 (382.2)	155.9–1735.4	472.8	391.8 (94.4)	243.9–597.7	365.4	0.2170
	E3	432.4 (282.9)	148.0–1553.4	387.4	404.8 (104.5)	232.1–619.4	411.2	0.5501
	E6	415.0 (208.5)	147.8–1032.4	396.5	392.1 (85.2)	249.0–567.6	388.0	0.9581
**Interleukin 6 [pg/mL]**	E0	9.0 (12.8)	1.2–56.8	4.2	2.7 (7.7)	0.2–43.9	1.0	0.0000 *
	E3	10.4 (5.9)	1.2–68.4	4.8	2.2 (5.6)	0.1–32.1	1.2	0.0000 *
	E6	9.0 (12.3)	1.1–51.6	5.2	2.4 (6.7)	0.3–38.3	1.1	0.0000 *

X—arithmetic mean; SD—standard deviation; Me—median; Range—(minimum–maximum); E0—measurement at study entry; E3—measurement after 3 months of the study; E6—measurement after 6 months of the study; *p*-value—statistical significance; *—statistically significant parameter; *n*–number.

**Table 3:** Analysis of correlations between the plasma levels of SCL and IL-6 within the study group and the control group and the body weight, BMI, and waist circumference of subjects (*R*—correlation coefficient).

Parameter		Sclerostin						Interleukin 6					
		Study Group (*n* = 34)			Control Group (*n* = 31)			Study Group (*n* = 34)			Control Group *(n* = 31)		
		E0	E3	E6	E0	E3	E6	E0	E3	E6	E0	E3	E6
**Body weight [kg]**	*R*	−0.10	−0.07	−0.01	0.26	0.36	0.26	0.03	0.05	0.03	0.25	0.36	0.49
	*p*-value	0.5723	0.6637	0.9781	0.1564	0.0443 *	0.1544	0.8529	0.7546	0.8651	0.1687	0.0480 *	0.0043 *
**BMI [kg/m^2^]**	*R*	−0.14	−0.16	−0.13	0.27	0.43	0.29	−0.06	−0.06	−0.07	0.33	0.46	0.53
	*p*-value	0.4560	0.3478	0.4774	0.1279	0.0156 *	0.1168	0.7254	0.7281	0.6745	0.0689	0.0097 *	0.0021 *
**Waist circumference [cm]**	*R*	−0.20	−0.07	−0.10	0.45	0.49	0.44	0.12	0.08	0.11	0.27	0.54	0.50
	*p*-value	0.2652	0.6856	0.5637	0.0106 *	0.0049 *	0.0112*	0.4949	0.6131	0.5452	0.1362	0.0016 *	0.0039 *
**Hip circumference [cm]**	*R*	−0.29	−0.27	−0.19	0.21	0.35	0.26	−0.03	−0.12	−0.17	0.24	0.52	0.41
	*p*-value	0.0854	0.1178	0.2789	0.2571	0.0522	0.1500	0.8546	0.4753	0.3331	0.1800	0.0025 *	0.0188 *
**WHR**	*R*	0.09	0.18	0.03	0.53	0.52	0.47	0.20	0.16	0.32	0.25	0.45	0.42
	*p*-value	0.960	0.2843	0.8442	0.0018 *	0.0026 *	0.0068 *	0.2526	0.3380	0.0574	0.1678	0.0099 *	0.0165 *

E0—measurement at study entry; E3—measurement after 3 months of study; E6—measurement after 6 months of study; *R*—correlation coefficient; *p*-value—statistical significance; *—statistically significant parameter; BMI—body mass index; WHR—waist hip ratio; *n*—number.

**Table 4:** Analysis of correlations between the plasma levels of SCL and IL-6 within the study group and the control group and the age of the subjects (*R*–correlation coefficient).

Parameter		Sclerostin		Interleukin 6	
		Study Group (*n* = 34)	Control Group (*n* = 31)	Study Group (*n* = 34)	Control Group (*n* = 32)
**E0**	*R*	0.14	0.34	0.08	0.46
	*p*-value	0.4018	0.0588	0.6570	0.0101 *
**E3**	*R*	0.53	0.48	0.02	0.52
	*p*-value	0.014 *	0.0058 *	0.9281	0.0026 *
**E6**	*R*	0.27	0.30	0.16	0.41
	*p*-value	0.1272	0.0915	0.3412	0.0205 *

E0—measurement at study entry; E3—measurement after 3 months of the study; E6—measurement after 6 months of the study; *R*—correlation coefficient; *p*-value—statistical significance; *—statistically significant parameter; *n*—number.
